# Supplementary material for: SLAMseq reveals potential transfer of RNA from liver to kidney in the mouse
Source: Nat Commun. 2025 Aug 11;16:7413. doi: 10.1038/s41467-025-62688-9 (PMC12339719; doi:10.1038/s41467-025-62688-9)
Supplement: Supplementary file 1 — Supplementary Information [file 41467_2025_62688_MOESM1_ESM.pdf]

SLAMseq reveals transfer of RNA from liver to kidney in the mouse

Supplementary Information

Supplementary information included in this .pdf:

- Supplementary Figures (1 – 17)
- Supplementary Tables (1 – 6)
- Supplementary Methods
- Supplementary References

Supplementary information uploaded in addition to this .pdf:

- Supplementary Data Files (1 – 13)
- Source data for all figures and tables

Supplementary Figures

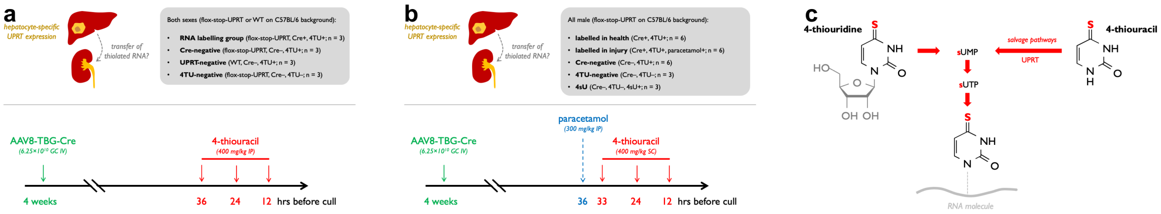

Supplementary Fig, 1. Experimental design. **a** Protocol for small initial experiment. An AAV8 vector was used to deliver Cre recombinase specifically to hepatocytes in floxed-stop-UPRT mice approximately 4 weeks prior to RNA labelling. Mice were dosed with 4-thiouracil (400 mg per kg) at 36, 24 and 12 hrs prior to cull. **b** Liver injury protocol. Mice in the injury group were dosed with paracetamol (300 mg per kg) at 36 hrs prior to cull; the first dose of 4-thiouracil was then given 3 hrs after paracetamol (i.e. 33 hrs prior to cull) and then at 24 and 12 hrs prior to cull. **c** Metabolism of 4TU and 4sU. The ribonucleoside 4sU, 4-thiouridine, is imported into all metazoan cells by equilibrative nucleoside transporters and incorporated into nascent RNA. 4-thiouracil, 4TU, must first be converted into thiolated uridine monophosphate (sUMP) before it can be incorporated into RNA. In higher eukaryotes, salvage pathways converting 4TU to sUMP operate with low efficiency[1]. The expression of recombinant protozoan UPRT permits the efficient conversion of 4TU to sUMP and thence incorporation into RNA molecules. Source data are provided as a Source Data file.

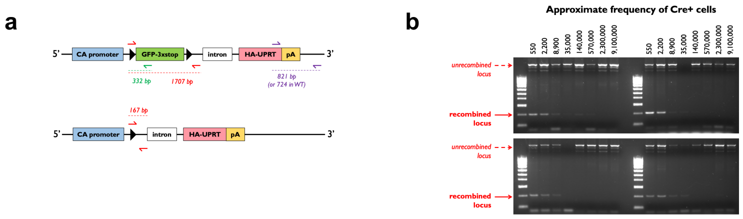

Supplementary Fig. 2. Genomic DNA (gDNA) recombination assay. a Design of recombination assay. Primers (red) were designed to amplify a 1707 bp region of the unrecombined flox-stop-UPRT locus or a 167 bp region of the recombined locus. The green reverse primer was used to amplify a 332 bp region only from the unrecombined locus and purple primer set for genotyping. **b** Recombination dilution series in podo-UPRT. gDNA was prepared from kidneys in podocin-UPRT mice and then used in a dilution series to estimate the sensitivity of the recombination assay. gDNA was diluted serially into gDNA from wild-type kidney and then used as a template in the PCR recombination assay reaction (n = 4). Assuming that podocytes constitute ~3% of all kidney cells and that Cre recombination in podocytes is 100% efficient, the approximate frequency of recombined cells is shown at the top of the figure. The assay was reliably able to detect Cre recombination, even when the recombined cells were estimated to constitute ~1 in 35,000 kidney cells and could detect recombination in some replicates down to an estimated frequency of 1 in 570,000 cells. Source data are provided as a Source Data file.

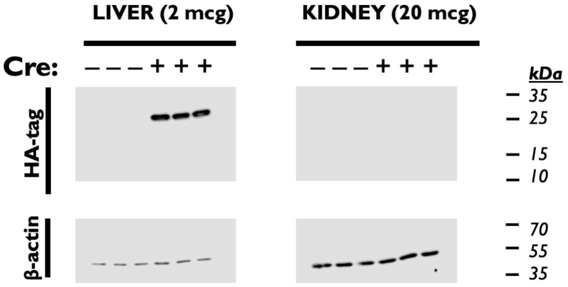

Supplementary Fig. 3. Immunoblot replicates. Replicate immunoblots (repeating the blot presented in Fig. 1d). Recombinant UPRT was detected by immunoblot (probing for the HA tag) in liver (2 micrograms of tissue homogenate per lane) and kidney (20 micrograms). β-actin was probed as a loading control. Cre-mediated UPRT expression was detected in liver but not kidney. Source data are provided as a Source Data file.

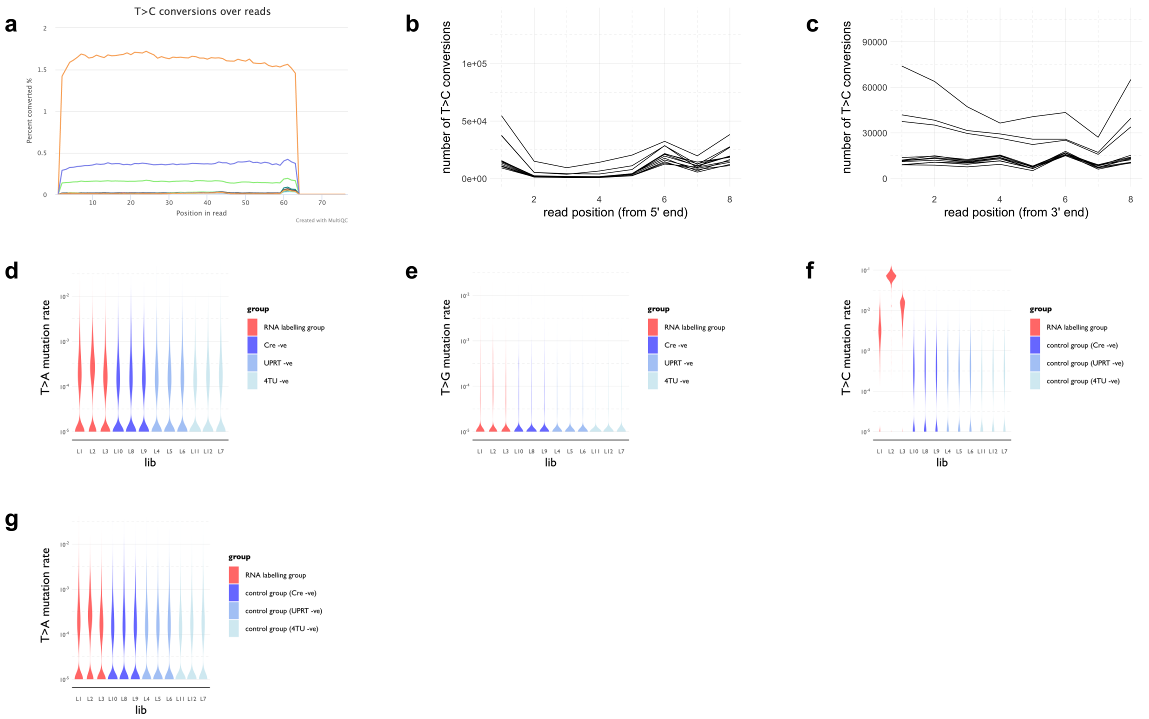

Supplementary Fig. 4. Initial labelling experiment: RNAseq characteristics. a T>C conversion rate by read position in mRNA data (export from multiqc analysis). **b&c** T>C conversion rate by read position in the small RNA data. **d** T>A conversion rates in mRNA data, per library. **e** T>G conversion rates in mRNA data, per library. **f** Correlation between T>C and T>A conversion rates in mRNA data. R^2 by linear regression = 0.012. **g** Correlation between T>C and T>G conversion rates in mRNA data. R^2 by linear regression = 0.042. Data derived from male and female mice, n = 3 in each experimental group. Source data are provided as a Source Data file.

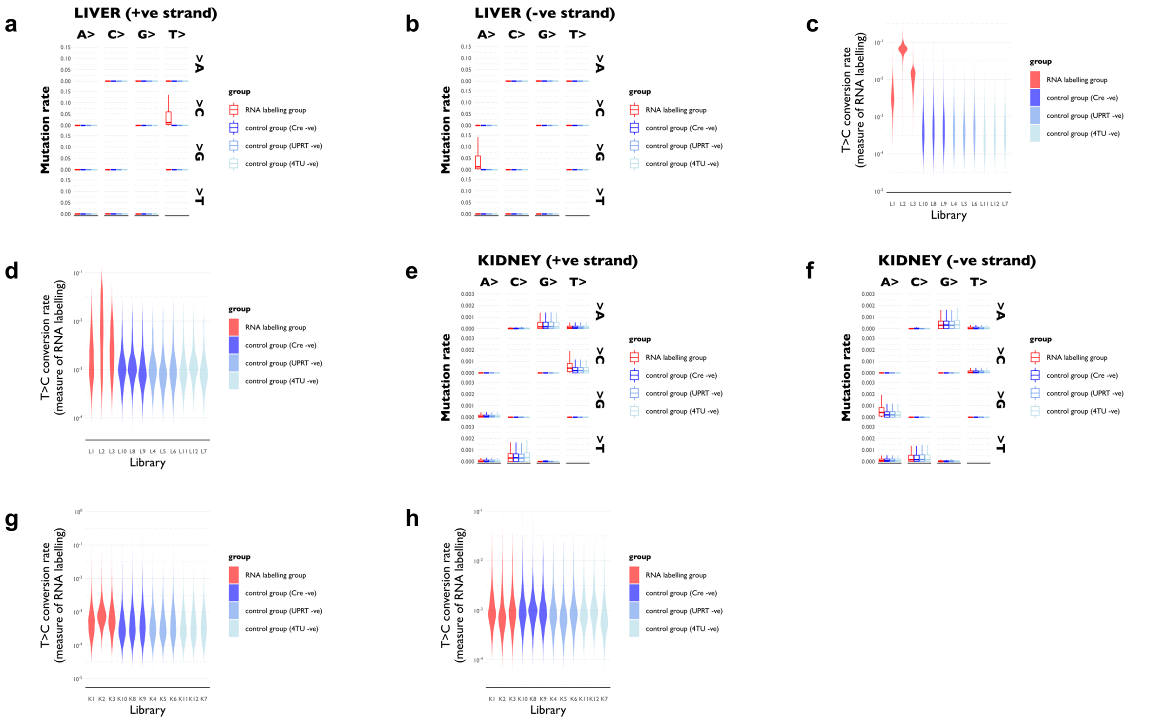

Supplementary Fig. 5. Initial labelling experiment: additional SLAMseq data. **a&b** Liver mRNA data: mutation rates for each possible nucleotide conversion. **c** Per library T>C conversion rates in liver mRNA. **d** Per library T>C conversion rates in liver small RNA. **e&f** Kidney mRNA data: mutation rates for each possible nucleotide conversion. **g** Per library T>C conversion rates in kidney mRNA. **h** Per library T>C conversion rates in kidney small RNA. Data derived from male and female mice, n = 3 in each experimental group. Source data are provided as a Source Data file.

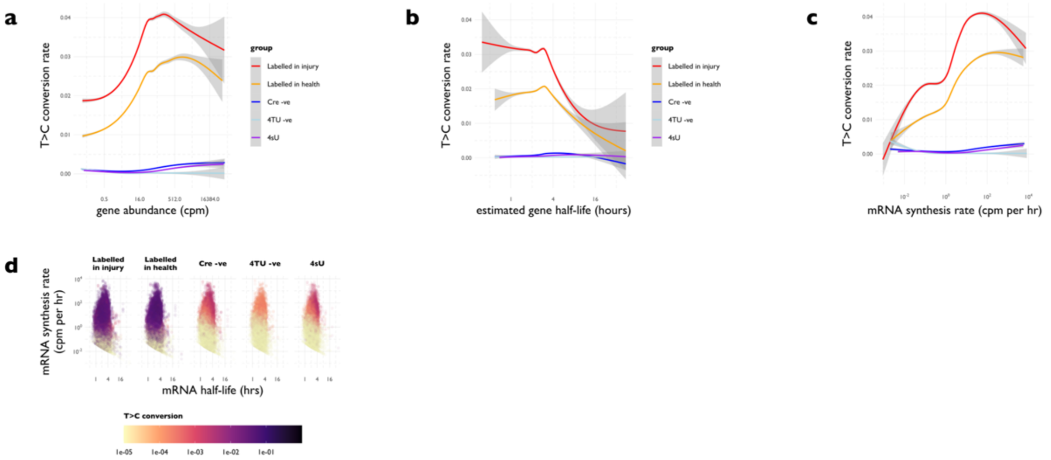

Supplementary Fig. 6. Relationship between T>C conversion rate, transcript abundance, half-life and synthesis rate. **a** Relationship between gene abundance (in cpm) and T>C conversion rate in mRNA data. **b** Relationship between estimated transcript half-life (imported from a published external dataset) and T>C conversion rate. **c** Relationship between estimated RNA synthesis rate and T>C. Gene-wise synthesis rate was computed from the observed abundance (cpm) and the estimated half-life (in published external dataset). **d** Same relationships in b and c, plotted in three dimensions using a colour-scale for T>C conversion rate. As synthesis rate increases so does T>C conversion rate (move vertically up the y-axis); as half-life increases, so does T>C conversion rate (move horizontally right along the x-axis). Data from male mice; n = 6 (labelled after paracetamol), n = 6 (labelled in health), n = 5 (Cre-negative control), n = 3 (4TU-negative control). Source data are provided as a Source Data file.

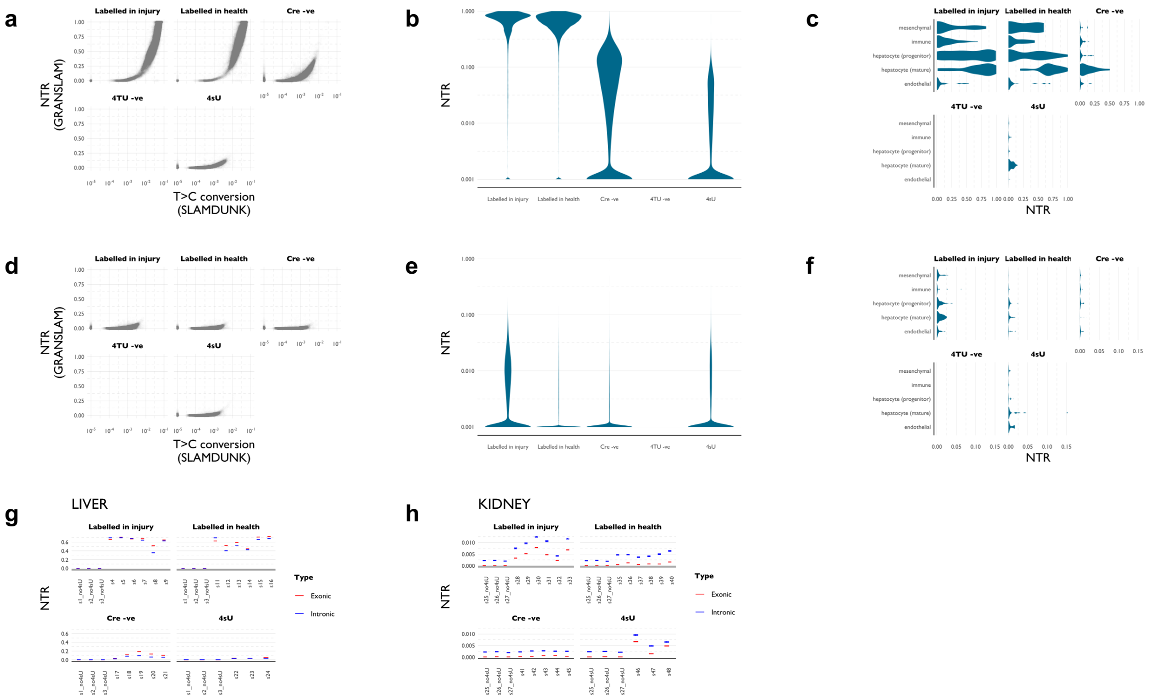

Supplementary Fig. 7. GRAND-SLAM analysis of T>C conversion rates in mRNA. a–c Liver mRNA. **d–f** Kidney mRNA. **a,d** Correlation between T>C conversion rates determined in the primary analysis (SLAMDUNK) and new-to-total RNA ratio (NTR): a metric of RNA labelling reported by GRAND-SLAM analysis. **b,e** Ratio of new-to-total (NTR) RNA as determined by GRAND-SLAM analysis of SLAMseq data. Violin plot shows the distribution of results in the different experimental groups. **c,f** NTR in marker genes, known to exhibit enriched expression in defined liver cell types. **g, h** NTR estimates from GRANDSLAM analysis of liver (g) and kidney (h) libraries. The error bars show the upper and lower bounds of the confidence interval. Each experimental group was compared to the same three 4sU-negative control libraries (represented for context in each of the four panels). Data from male mice; n = 6 (labelled after paracetamol), n = 6 (labelled in health), n = 5 (Cre-negative control), n = 3 (4TU-negative control). Source data are provided as a Source Data file.

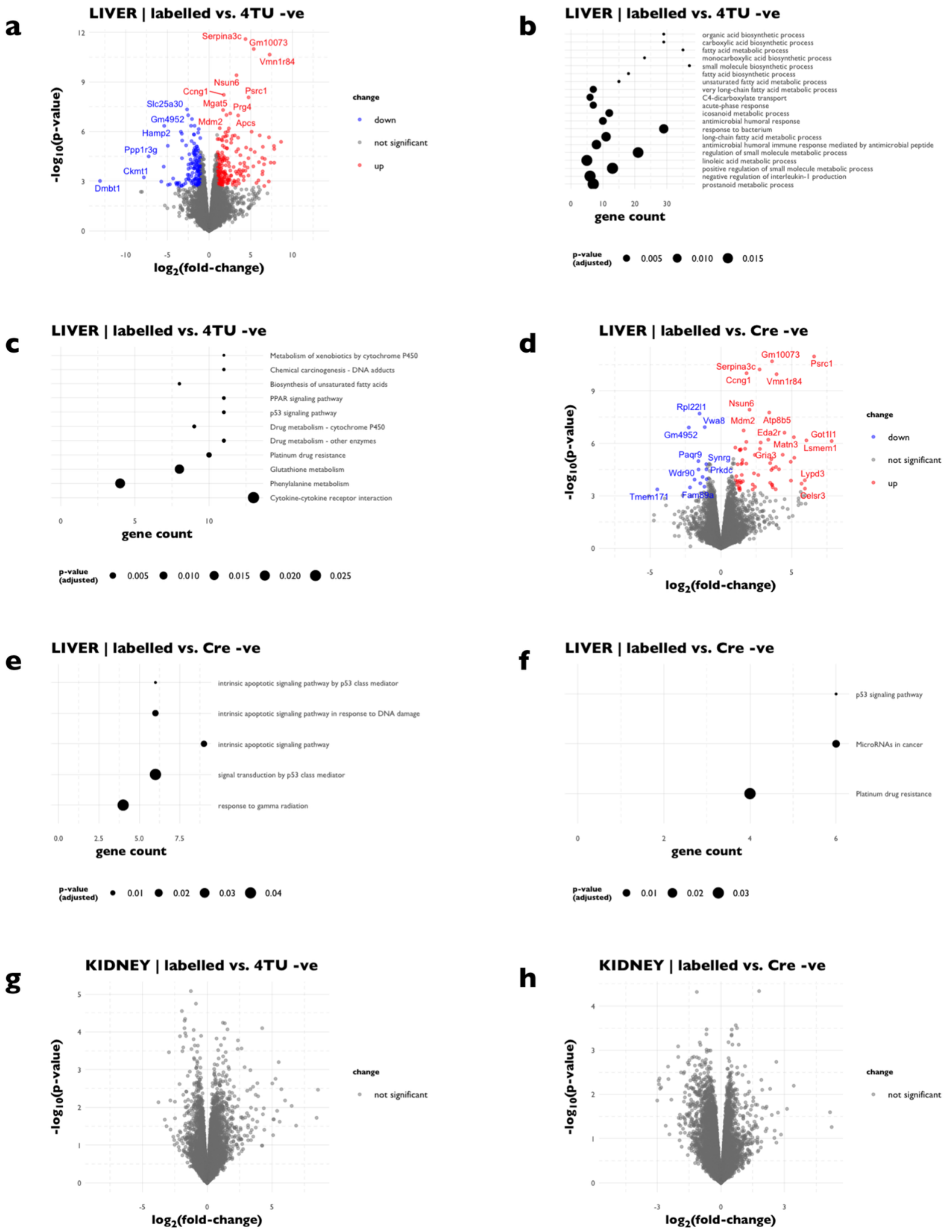

Supplementary Fig. 8. Effects of the RNA labelling protocol on gene expression. a Differential expression analysis of liver mRNA data, showing genes up-regulated (red) or down-regulated (blue) in the "RNA Labelled in health" group compared to the unlabelled 4TU-negative group, setting significant false discovery rate at 0.05. This negative control group was not exposed to AAV8-TBG-Cre or 4TU. **b, c** Gene ontology (b) and KEGG (c) analyses of the genes differentially expressed in a. **d** Differential expression analysis of liver mRNA data, showing genes up-regulated (red) or down-regulated (blue) in the "RNA Labelled in health" group compared to the Cre-negative group. This negative control group was exposed to 4TU but not AAV8-TBG-Cre. **e, f** Gene ontology (e) and KEGG (f) analyses of the genes differentially expressed in d. **g,h** Differential expression analysis of kidney mRNA data. There were no differentially expressed genes in the RNA labelling group compared to either of the negative control groups. Data from male mice; n = 6 (labelled after paracetamol), n = 6

(labelled in health), n = 5 (Cre-negative control), n = 3 (4TU-negative control). Source data are provided as a Source Data file.

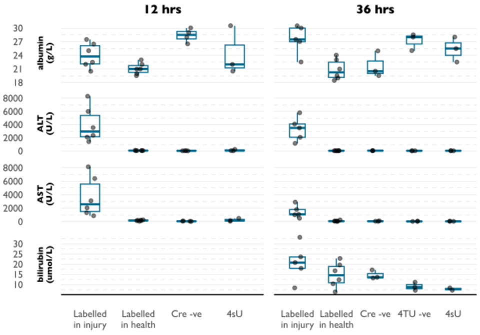

Supplementary Fig. 9. Liver function tests in the liver injury experiment. Plasma concentrations of ALT, AST, albumin and total bilirubin were determined at 12 and 36 hours after paracetamol administration. Data from male mice; n = 6 (labelled after paracetamol), n = 6 (labelled in health), n = 5 (Cre-negative control), n = 3 (4TU-negative control). Source data are provided as a Source Data file.

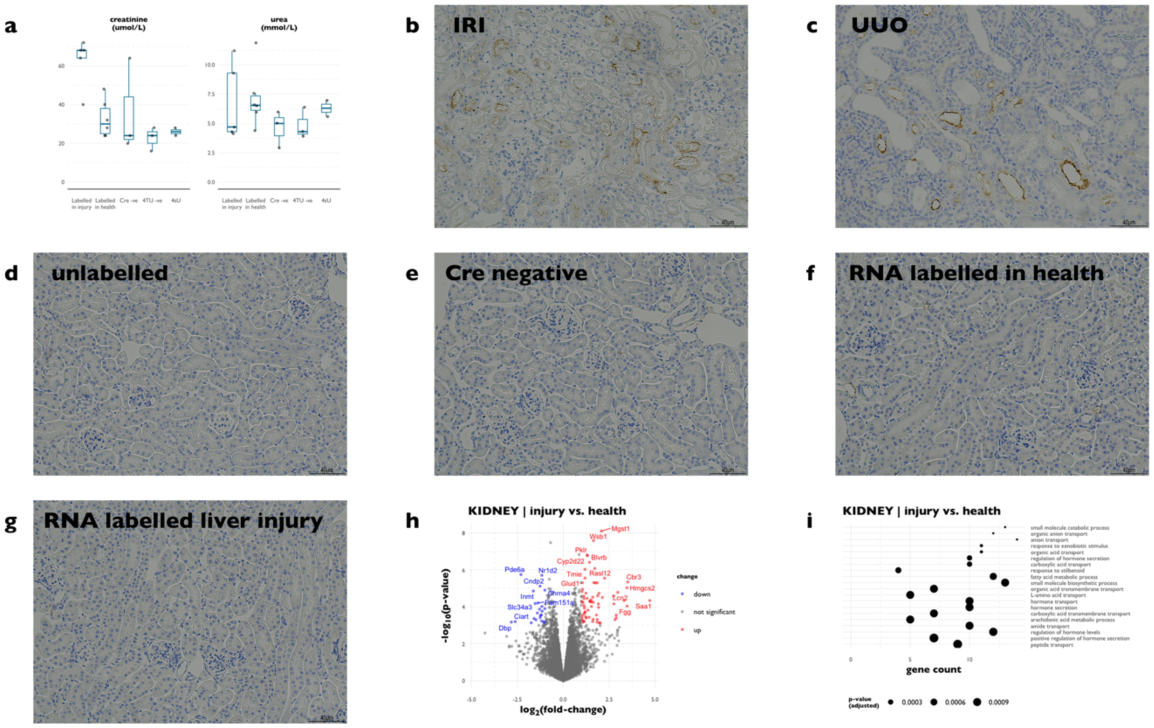

Supplementary Fig. 10. Effects of paracetamol on kidney structure and function. a Plasma concentrations of urea and creatinine, measured 36 hours after paracetamol. **b-g** Immunohistochemistry for Kidney Injury Molecule 1 (KIM-1). Positive control kidneys taken from mouse kidneys exposed to ischaemic-reperfusion injury (IRI) or unilateral ureteric obstruction (UUO) were used to validate KIM1 immunodetection. In those injured kidneys, KIM-1 was detected in the apical membranes of renal tubular epithelial cells (b&c). In contrast, KIM-1 was not detected in any of the control or experimental groups in the RNA labelling experiment. There were no histological abnormalities evident in these kidneys either (see also H&E and PAS stains in Supplementary Data File 7). Data from male mice; n = 6 (labelled after paracetamol), n = 6 (labelled in health), n = 5 (Cre-negative control), n = 3 (4TU-negative control). **h&i** Volcano plot & GO analysis showing genes differentially expressed in the kidney after paracetamol-induced liver injury. Source data are provided as a Source Data file.

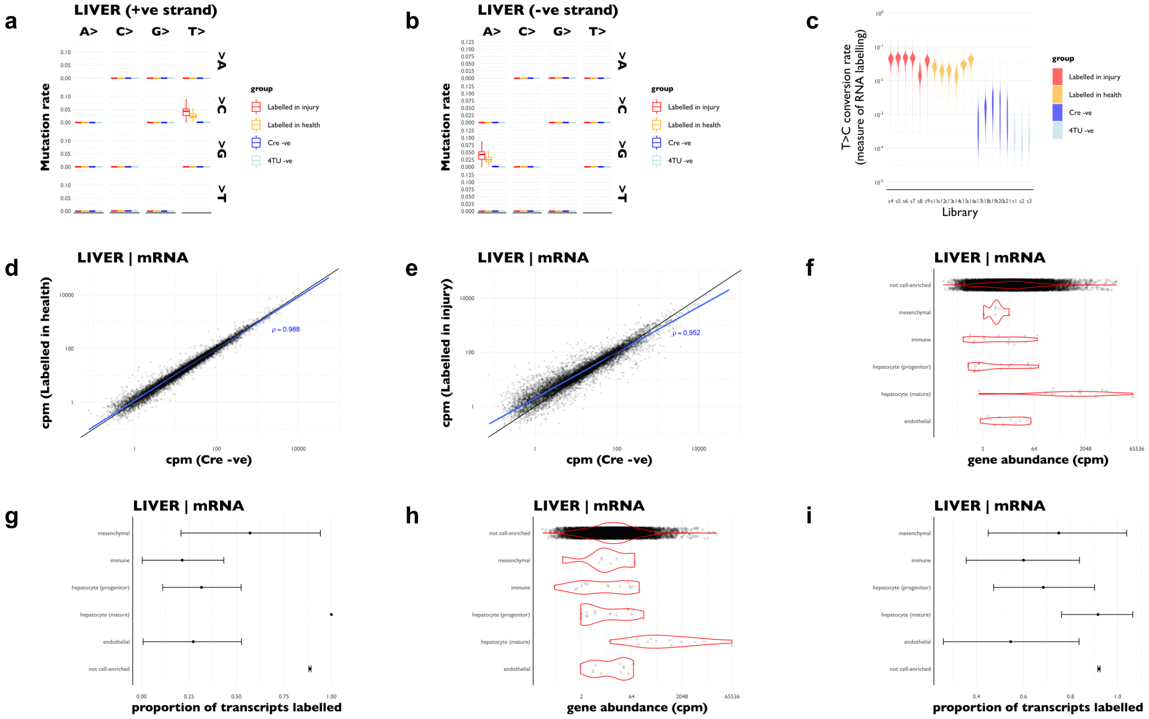

Supplementary Fig. 11. Liver injury experiment: additional SLAMseq liver mRNA data. a&b Nucleotide conversion rates. **c** Per library T>C conversion rates. **d** Correlation in gene abundance (cpm) between 'Labelled in health' and 'Cre-negative' groups; these data were analysed in the beta-binomial test presented in Fig. 5b. **e** Correlation in gene abundance (cpm) between 'Labelled in injury' and 'Cre-negative' groups; these data were analysed in the beta-binomial test presented in Fig. 5c. **f&g** Marker genes in 'Labelled in health' group. RNA labelling was assessed in genes known to be enriched in defined liver cell types. Gene abundance (cpm) shown in f; proportion of transcripts labelled with T>C conversions shown in g. **h&i** Marker genes in 'Labelled in injury' group. Error bars depict 95% confidence intervals. Source data are provided as a Source Data file.

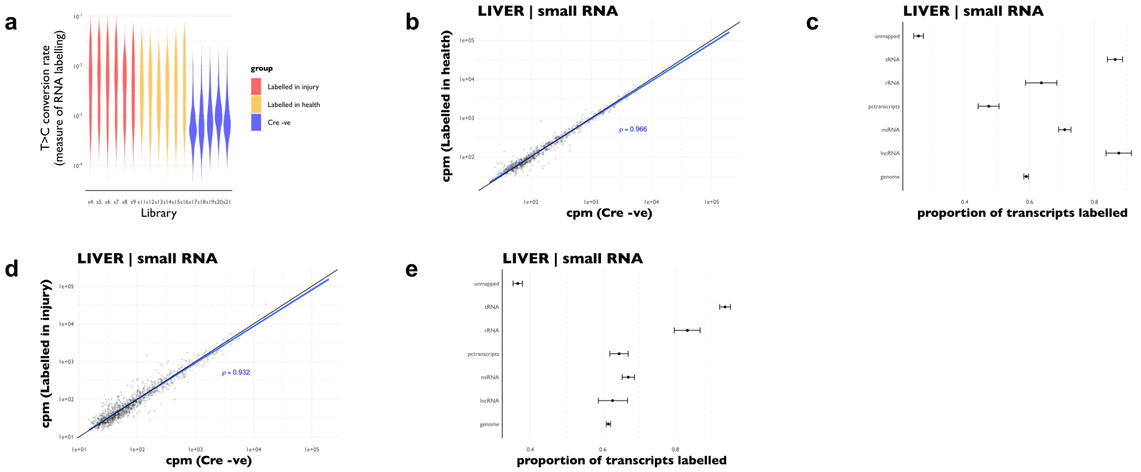

Supplementary Fig. 12. Liver injury experiment: additional SLAMseq liver small RNA data. a Per library T>C conversion rates. **b** Correlation in gene abundance (cpm) between 'Labelled in health' and 'Cre-negative' groups; these data were analysed in the beta-binomial test presented in Fig. 5f. **c** RNA labelling in the 'Labelled in health' group, stratified by small RNA biotype. **d** Correlation in gene abundance (cpm) between 'Labelled in injury and 'Cre-negative' groups; these data were analysed in the beta-binomial test presented in Fig. 5g. **e** RNA labelling in the 'Labelled in health' group, stratified by small RNA biotype. Error bars depict 95% confidence intervals. Source data are provided as a Source Data file.

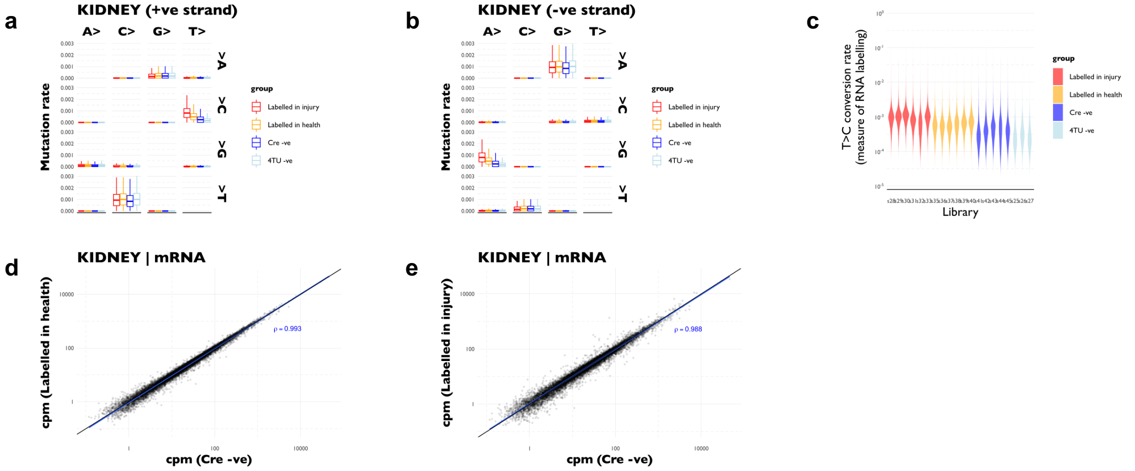

Supplementary Fig. 13. Liver injury experiment: additional SLAMseq kidney mRNA data. a&b Nucleotide conversion rates. **c** Per library T>C conversion rates. **d** Correlation in gene abundance (cpm) between 'Labelled in health' and 'Cre-negative' groups; these data were analysed in the beta-binomial test presented in Fig. 6b. **e** Correlation in gene abundance (cpm) between 'Labelled in injury' and 'Cre-negative' groups; these data were analysed in the beta-binomial test presented in Fig. 6c. Source data are provided as a Source Data file.

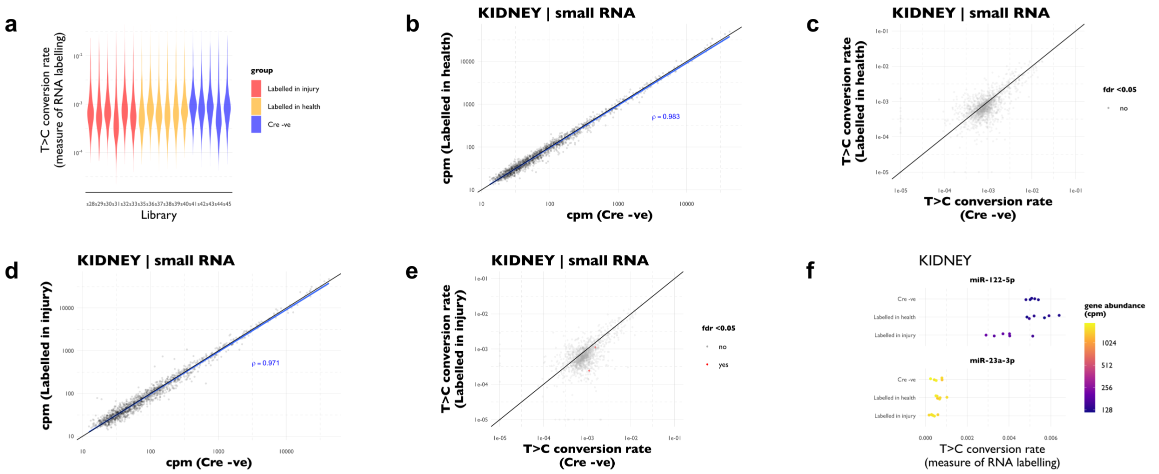

Supplementary Fig. 14. Liver injury experiment: additional SLAMseq kidney small RNA data. **a** Per library T>C conversion rates. **b&c** Gene-wise comparison between 'Labelled in health' and 'Cre-negative' groups, showing gene abundance in b and results of beta-binomial test for differences in T>C conversions in c. **d&e** Gene-wise comparison between 'Labelled in injury and 'Cre-negative' groups, showing gene abundance in d and results of beta-binomial test for differences in T>C conversions in e. **f** T>C conversion rates in the known hepatocyte-enriched miRNA, miR-122 and the known endothelial-enriched miRNA, miR-23a. Source data are provided as a Source Data file.

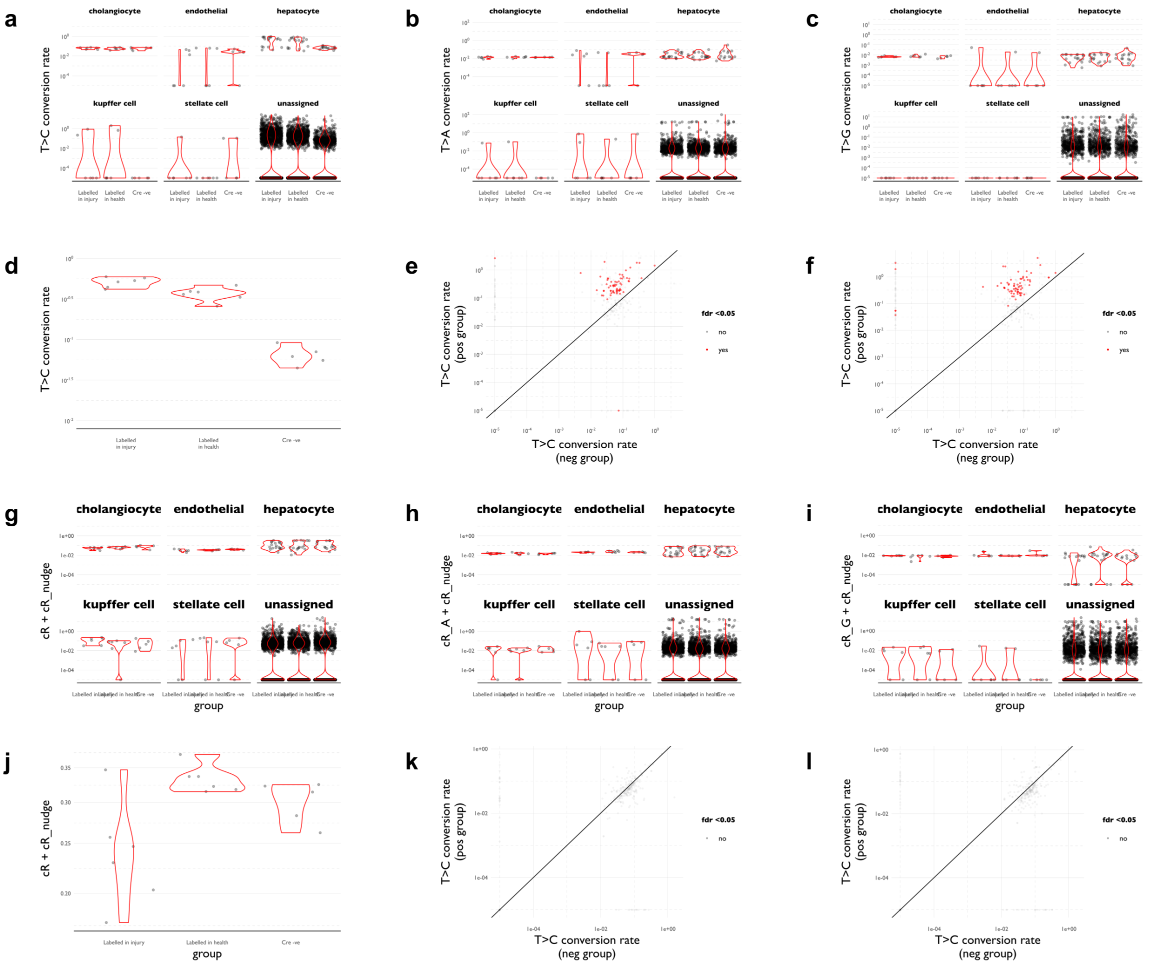

Supplementary Fig. 15. Re-analysis of small RNA data using alternative mapping approach. The small RNA SLAMseq data were re-analysed using an alternative strategy, using a single mapping step to the whole genome. **a**-c T>C, T>A and T>G conversion rates in the liver, within miRNAs known to be enriched in defined cell types. Elevated rates of T>C conversion were observed in RNA labelling groups in miRNAs known to be expressed in hepatocytes and those not enriched in any of the defined cells; rates of T>A and T>G conversions were not affected. **d** T>C conversions in the known hepatocyte-enriched miRNA, miR-122 in liver. **e&f** Beta-binomial tests, identifying labelled miRNAs in the "Labelled in health" (e) and "Labelled in injury" (f) groups. **g-l** The same analyses in kidney sRNA data. There was no increase in T>C conversion rate in hepatocyte or unassigned miRNAs, or in miR-122. No miRNAs were identified as being labelled in kidney tissue. Source data are provided as a Source Data file.

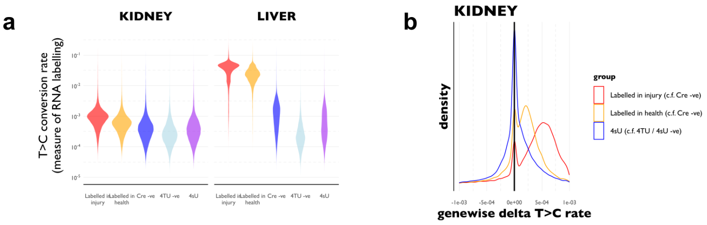

Supplementary Fig. 16. RNA labelling with 4-thiouridine (4sU). a The 4sU administration protocol induced a low level of T>C conversion in liver and kidney, approximately equivalent to that seen in the Cre-negative control group. Data are the same as in Figs. 5a & 6a, with the addition of the 4sU group. **b** Gene-wise differences in T>C conversion rates. The difference in T>C conversion rate between labelling and negative control groups were determined for each gene. The relevant control group was the “Cre-negative”, 4TU-exposed group for the “Labelled in health” and “Labelled in injury” groups and the 4sU, 4TU-naïve, “4TU-negative” group for the “4sU” group. Source data are provided as a Source Data file.

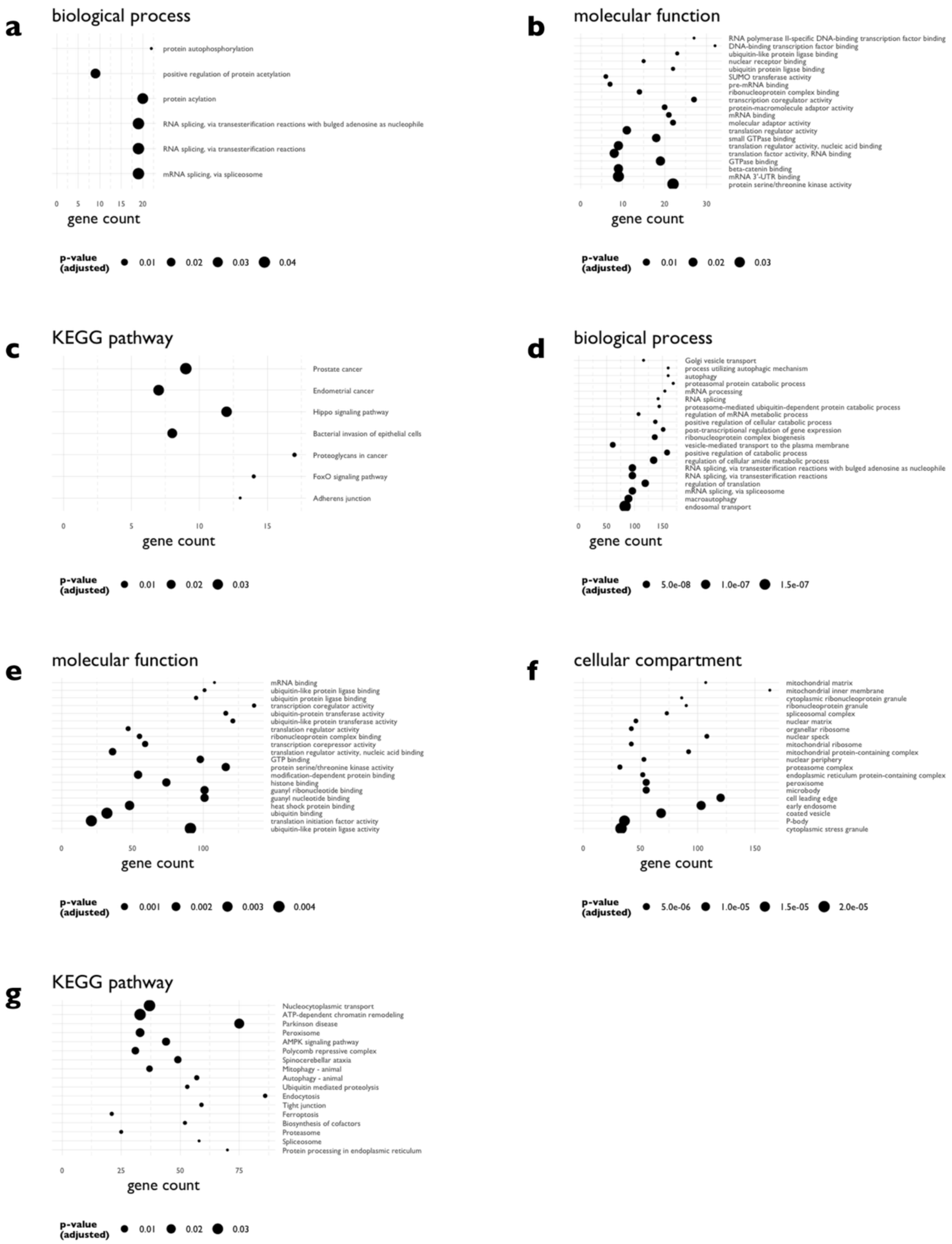

Supplementary Fig. 17. Pathway analyses of mRNAs likely to be transferred from liver to kidney. a-c Gene ontology (**a, b**) and KEGG pathway (**c**) analyses of those mRNAs that were labelled in the kidney in both health and liver injury in 4TU-treated mice, but not in 4sU-treated mice (i.e. the set containing 478 mRNAs in Fig. 7f). d-g GO (**d-f**) and KEGG (**g**) analyses of those mRNAs that were labelled in the kidney exclusively in liver injury (i.e. the set containing 3690 mRNAs in Fig. 7f). In these analyses, the set of labelled mRNAs was compared to a "background" set comprising all of the mRNAs present in the corresponding kidney RNAseq dataset. Source data are provided as a Source Data file.

Supplementary Tables

animal	sex	age	strain	AAV8-Cre	4TU	group
S1	m	9m 22d	floxed-stop-UPRT	1	1	RNA labelling
S2	m	8m 18d	floxed-stop-UPRT	1	1	RNA labelling
S3	f	8m 18d	floxed-stop-UPRT	1	1	RNA labelling
S4	m	4m 24d	wild-type	0	1	UPRT neg
S5	m	4m 24d	wild-type	0	1	UPRT neg
S6	m	4m 24d	wild-type	0	1	UPRT neg
S7	m	4m 22d	floxed-stop-UPRT	0	0	4TU neg
S8	m	4m 30d	floxed-stop-UPRT	0	1	Cre neg
S9	m	4m 30d	floxed-stop-UPRT	0	1	Cre neg
S10	m	4m 21d	floxed-stop-UPRT	0	1	Cre neg
S11	m	4m 21d	floxed-stop-UPRT	0	0	4TU neg
S12	m	4m 21d	floxed-stop-UPRT	0	0	4TU neg

Supplementary Table 1. Experimental animals in the initial RNA labelling experiment. 1 denotes treatment received; 0 denotes treatment not received.

animal	sex	age (days)	group	Cre	APAP	4TU	4sU
1	m	67	Unlabelled	0	0	0	0
2	m	108	Unlabelled	0	0	0	0
3	m	102	Unlabelled	0	0	0	0
4	m	100	Labelled in injury	1	1	1	0
5	m	100	Labelled in injury	1	1	1	0
6	m	100	Labelled in injury	1	1	1	0
7	m	100	Labelled in injury	1	1	1	0
8	m	165	Labelled in injury	1	1	1	0
9	m	165	Labelled in injury	1	1	1	0
11	m	186	Labelled in health	1	0	1	0
12	m	186	Labelled in health	1	0	1	0
13	m	148	Labelled in health	1	0	1	0
14	m	148	Labelled in health	1	0	1	0
15	m	95	Labelled in health	1	0	1	0
16	m	95	Labelled in health	1	0	1	0
17	m	114	Cre neg	0	0	1	0
18	m	245	Cre neg	0	0	1	0
19	m	245	Cre neg	0	0	1	0
20	m	67	Cre neg	0	0	1	0
21	m	67	Cre neg	0	0	1	0
22	m	77	4sU	0	0	0	1
23	m	77	4sU	0	0	0	1
24	m	67	4sU	0	0	0	1

Supplementary Table 2. Experimental animals in the liver injury experiment. 1 denotes treatment received; 0 denotes treatment not received.

	T>C conversion % (median, IQR)			
	**mRNA**		**small RNA**	
**group**	*liver*	*kidney*	*liver*	*kidney*
RNA labelling	0.709 (0.043 - 4.474) **	0.009 (0.000 - 0.063) **		
Cre-negative	0.000 (0.000 - 0.027)	0.000 (0.000 - 0.032)		
UPRT-negative	0.000 (0.000 - 0.030) *	0.000 (0.000 - 0.031)		
4TU-negative	0.000 (0.000 - 0.021) **	0.000 (0.000 - 0.030) **		

Supplementary Table 3. T>C conversion rates in initial experiment. Rates are expressed as % - i.e. a rate of 1% means conversion of 1 in 100 Ts. * $p = 0.0002$; ** $p < 10^{-9}$ for comparison to the Cre-negative group within that tissue by two-sided Wilcoxon signed rank test after Kruskal-Wallis rank sum test. Source data are provided as a Source Data file.

	**mRNA**			**small RNA**		
**tissue**	*not labelled*	*labelled*	%	*not labelled*	*labelled*	%
**liver**	10,451	1,501	12.6	1,291	327	20.2
**kidney**	13,500	81	0.6	1,265	0	0.0

Supplementary Table 4.RNA labelling rates in initial experiment. The number of mRNAs and small RNAs (i.e. distinct genes) labelled in liver and kidney in the RNA labelling group. Source data are provided as a Source Data file.

**experiment**	**RNAseq modality**	**reads per trimmed library** (mean ± SD)	**reads used for T>C quantification** (mean ± SD)
initial experiment in male and female mice	*mRNA (Quantseq)*	21.7 ± 1.71 million	10.1 ± 0.81 million
	small RNA	22.5 ± 5.7 million	18.5 ± 5.1 million
liver injury in male mice	*mRNA (Quantseq)*	26.1 ± 3.68 million	15.1 ± 2.02 million
	small RNA	17.2 ± 3.1 million	15.1 ± 2.8 million

Supplementary Table 5. RNAseq read counts. The number of reads used to T>C quantification, after mapping, filtering and excluding SNPs, was smaller than the total number of reads in trimmed libraries.

RNA biotype	taxonomy	database
miRNA	10090	miRBase (release 22)
pre-miRNA	10090	miRBase (release 22)
rRNA	10090	Ensembl (release 109)
tRNA	10090	ENA (as of Feb 2023)
snoRNA	10090	Ensembl (release 109)
YRNA	10090	Ensembl (release 109)
piRNA	10090	pirBase (release v3.0)
lncRNA	10090	RefSeq (release 213)
snRNA	10090	Ensembl (release 109)
vaultRNA	10090	Ensembl (release 109)

Supplementary Table 6. Databases used for mapping small RNA.

Supplementary Methods

Biochemical assays

All biochemical assays were performed using commercial kits, adapted for use on a Cobas Fara analyser (Roche Diagnostics Ltd, Welwyn Garden City, UK).

Plasma alanine transaminase (ALT) activity was measured in a kinetic assay,[2] using a kit (17234H) from Sentinel Diagnostics via Alpha Laboratories Ltd., Eastleigh, UK. Intra-assay and inter-assay precision were CV < 4% and CV < 8% respectively.

Aspartate aminotransferase (AST) activity was determined using a kit (17224H) from Sentinel Diagnostics. Intra-assay and inter-assay precision was CV <4% and CV < 5% respectively.

Total bilirubin concentration was determined by the acid diazo method[3] using a kit (BR243) from Randox Laboratories, County Antrim, UK. Intra-assay precision was CV < 4%; inter-assay precision was CV < 5%.

Albumin concentration was determined using a kit (17600H) from Sentinel Diagnostics. Intra-assay precision was CV < 2.5%; inter-assay precision was CV < 5%.

Urea concentration was determined using a kit (17629H) from Sentinel Diagnostics. Intra-assay precision was CV < 3%; inter-assay precision was CV < 5%.

Creatinine concentration was determined using a creatininase/creatinase enzymatic method as described by Börner et al.[4], using a kit (17654H) from Sentinel Diagnostics. Intra-assay precision was < 3%; inter-assay precision was CV < 5.

Command line code for SLAMDUNK analysis

```
slamdunk map -t 18 -r
mmu_GRCm38.92_ameres_slamseq/annotation_organism_ercc_sirv.fa -5 12
-a 4 -n 100 -ss -o 2935663/tmp/ sample_sheet.tsv
```

```
samtools view -H 2935663/tmp/R1.fastq_slamdunk_mapped.bam | sed
's/ID:[0-9]/ID:24/' | samtools reheader -
2935663/tmp/R1.fastq_slamdunk_mapped.bam >
2935663/output/R1.fastq_slamdunk_mapped.bam
```

```
slamdunk filter -t 4 -b
mmu_GRCm38.92_ameres_slamseq/annotation_organism_UTR.bed -o
2935687/output/ 2935663/output/R1.fastq_slamdunk_mapped.bam
```

```
slamdunk snp -t 6 --var-fraction 0.2 -r
mmu_GRCm38.92_ameres_slamseq/annotation_organism_ercc_sirv.fa -o
2935711/output/ 2935687/output/R1.fastq_slamdunk_mapped_filtered.bam
```

```
slamdunk count -t 4 -b
mmu_GRCm38.92_ameres_slamseq/annotation_organism_UTR.bed -r
mmu_GRCm38.92_ameres_slamseq/annotation_organism_ercc_sirv.fa -o
2935735/output/ -s 2935711/output --max-read-length 101
2935687/output/R1.fastq_slamdunk_mapped_filtered.bam
```

```
alleyoop collapse -t 4 -o 2935735/output/
2935735/output/R1.fastq_slamdunk_mapped_filtered_tcount.tsv
```

```
alleyoop rates -t 4 -o 2935735/output/ -r
mmu_GRCm38.92_ameres_slamseq/annotation_organism_ercc_sirv.fa
2935687/output/R1.fastq_slamdunk_mapped_filtered.bam
```

```
alleyoop utrrates -t 4 -o 2935735/output/ -r
mmu_GRCm38.92_ameres_slamseq/annotation_organism_ercc_sirv.fa -b
mmu_GRCm38.92_ameres_slamseq/annotation_organism_UTR.bed --max-read-
length 101 2935687/output/R1.fastq_slamdunk_mapped_filtered.bam
```

```
alleyoop tcperreadpos -t 4 -o 2935735/output/ -r
mmu_GRCm38.92_ameres_slamseq/annotation_organism_ercc_sirv.fa -s
2935711/output --max-read-length 101
2935687/output/R1.fastq_slamdunk_mapped_filtered.bam
```

```
alleyoop tcperutrpos -t 4 -o 2935735/output/ -r
mmu_GRCm38.92_ameres_slamseq/annotation_organism_ercc_sirv.fa -s
2935711/output -b
mmu_GRCm38.92_ameres_slamseq/annotation_organism_UTR.bed --max-read-
length 101 2935687/output/R1.fastq_slamdunk_mapped_filtered.bam
```

Alternative approach to small RNA mapping

In a sensitivity analysis, we took an alternative approach to mapping smallRNAseq data. Instead of mapping sequentially to many small RNA genomes, we used Bowtie (v1.3.1) to map in a single step to the mouse genome (GRCm39). The commandline code for this was:

```
parallel "bowtie -t --threads $n_cores -q -v 1 -m 1 --all --best --
strata -x $mouse_index {} > {.}.uni.map" ::: *.fq
```

Output files were then annotated with a .gff file, constructed using the biomaRt R package (v2.54.1) to pull miRNA genes from ensemble.

GRAND-SLAM analysis

As an alternative way of quantifying T>C conversions in our mRNA SLAMSeq data, we used the GRAND-SLAM pipeline.[5] In this approach, for a given gene, one first estimates p_e (rate of erroneous T>C conversions) and p_c (rate of overall T>C conversions including incorporation rate, conversion rate and error rate). These are then used to estimate the proportion of new-to-total RNA for that gene (NTR).

We implemented this, following the instructions at https://github.com/erhard-lab/gedi/wiki/GRAND-SLAM, making use of our 4TU-negative control samples to help define p_e. The commands used were:

```
gedi -e IndexGenome -organism mus_musculus -version 92 -p -nobowtie
-nostar -nokallisto

parallel 'samtools sort {} -o {.}_sorted.bam' ::: *.bam

parallel 'samtools index {}' ::: *_sorted.bam

parallel "gedi -e Slam -genomic mus_musculus.92 -prefix {.} -D -
reads {}" ::: *.bamlist
```

Estimation of mRNA half-life and synthesis rates

To determine the relationship between T>C conversion and mRNA half-life / synthesis rate, we merged our mRNAseq data with external half-life data. Transcript half-life ($t_{½}$) was taken from a published dataset (supplemental table S1 from Agarwal & Kelly, 2022[6]). RNA

synthesis rate, k_s, in cpm per hour was estimated using the following equation: $k_s = \ln(2) * R / t_{½}$, where R was the abundance in cpm (measured from our RNAseq data) and $t_{½}$ was the transcript half-life in hours (imported from the external dataset). This equation assumes that mRNA levels are in steady state and $t_{½}$ is constant.

Quantification of T>C conversions in liver- vs. kidney-enriched genes

Within those genes that were present in both liver and kidney RNAseq data, we attempted to test whether T>C conversion rates were disproportionately higher in those kidney transcripts that were expressed at higher levels within liver.

In a differential expression analysis, we first defined genes as being liver-enriched, or kidney-enriched. After filtering out low abundance transcripts and normalising by the TMM (trimmed mean of M-values) method, we used the glmTreat() function in edgeR (v3.40.2) to identify genes differentially expressed in liver *vs*. kidney, setting a log-fold-change threshold of 5.

It would be unfair to compare crude T>C conversion rates within these gene sets within kidney tissue because, unsurprisingly, the kidney-enriched genes had higher average expression within kidney tissue and would therefore be expected to have higher rates of T>C conversion. Therefore, we pulled genes at random from these two sets (liver-enriched and kidney-enriched) within defined cpm bins, so deriving two sets of genes that shared a similar abundance profile within kidney RNAseq data but differed in the extent to which they were expressed in liver. To do this, we first summarised the expression of each gene in the kidney within each treatment group using the edgR::cpmByGroup() function. The distribution of cpms for liver-enriched genes was used to define 8 cpm bins containing approximately equal numbers of genes. Genes were then sampled at random from the set of liver- and kidney-enriched genes within each of the cpm bins, to approximate the cpm distribution of liver-enriched genes. T>C conversion rates were computed within the liver- and kidney-enriched gene sets. This sampling process was repeated 100 times, to allow the construction of confidence intervals around the point estimate for T>C conversion rate.

In a subsequent analysis, this process was repeated after first excluding any gene that exhibited 4sU-dependent labelling. This was defined as those genes in the top tertile of T>C conversion rates in the 4sU-treated group.

Supplementary References

1. Nainar, S. *et al.* An optimized chemical-genetic method for cell-specific metabolic labeling of RNA. *Nat. Methods* **17**, 311–318 (2020).

2. Bergmeyer, H. U., Scheibe, P. & Wahlefeld, A. W. Optimization of methods for aspartate aminotransferase and alanine aminotransferase. *Clin. Chem.* **24**, 58–73 (1978).

3. Pearlman, F. C. & Lee, R. T. Detection and measurement of total bilirubin in serum, with use of surfactants as solubilizing agents. *Clin. Chem.* **20**, 447–453 (1974).

4. Börner, U., Szász, G., Bablok, W. & Busch, E. W. [A specific fully enzymatic method for creatinine: reference values in serum (author's transl)]. *J. Clin. Chem. Clin. Biochem. Z. Klin. Chem. Klin. Biochem.* **17**, 679–682 (1979).

5. Jürges, C., Dölken, L. & Erhard, F. Dissecting newly transcribed and old RNA using GRAND-SLAM. *Bioinformatics* **34**, i218–i226 (2018).

6. Agarwal, V. & Kelley, D. R. The genetic and biochemical determinants of mRNA degradation rates in mammals. *Genome Biol.* **23**, 245 (2022).
