# Supplementary material for: SLAMseq reveals potential transfer of RNA from liver to kidney in the mouse
Source: Nat Commun. 2025 Aug 11;16:7413. doi: 10.1038/s41467-025-62688-9 (PMC12339719; doi:10.1038/s41467-025-62688-9)
Supplement: Supplementary file 2 — Description of Additional Supplementary Files [file 41467_2025_62688_MOESM2_ESM.pdf]

Description of Additional Supplementary Files

File Name: Supplementary Data 1
Description: replicate immunofluorescent images from mTmG reporter mouse (+/- AAV8-Tbg-Cre)

File Name: Supplementary Data 2
Description: metadata accompanying all RNAseq data

File Name: Supplementary Data 3
Description: multiQC files for all RNAseq data

File Name: Supplementary Data 4
Description: lists of labelled RNAs in the initial experiment

File Name: Supplementary Data 5
Description: beta binomial test outputs from initial experiment

File Name: Supplementary Data 6
Description: replicate H&E images of liver (liver injury experiment)

File Name: Supplementary Data 7
Description: replicate PAS and KIM1 immunohistochemistry images of kidney (liver injury experiment)

File Name: Supplementary Data 8
Description: lists of labelled RNAs in the liver injury experiment

File Name: Supplementary Data 9
Description: beta binomial test outputs from the liver injury experiment

File Name: Supplementary Data 10
Description: differential expression analysis of kidney miRNAs in liver injury experiment

File Name: Supplementary Data 11
Description: marker genes

File Name: Supplementary Data 12
Description: full GO and KEGG outputs from analysis of RNAs likely transferred from liver to kidney

File Name: Supplementary Data 13
Description: binomEstimated.tsv files from GRANDSLAM analysis – combined into a single table
