# Supplementary material for: SLAMseq reveals potential transfer of RNA from liver to kidney in the mouse
Source: Nat Commun. 2025 Aug 11;16:7413. doi: 10.1038/s41467-025-62688-9 (PMC12339719; doi:10.1038/s41467-025-62688-9)
Supplement: Supplementary file 6 — Source Data [file 41467_2025_62688_MOESM6_ESM.zip › Source data/FigS4a_data.html]

MultiQC Report


# Toggle navigation v1.0.dev0

- General Stats
- Slamdunk
  - Filter statistics
  - PCA (T>C based)
  - Conversion rates per UTR
  - Conversion rates per read
  - Non T>C mutations over read positions
  - T>C conversions over read positions
  - Non T>C mutations over UTR positions
  - T>C conversions over UTR positions

Toolbox

### MultiQC Toolbox

#### Apply Highlight Samples

+

Regex mode off
help
 Clear

#### Apply Rename Samples

+

Click here for bulk input.

Paste two columns of a tab-delimited table here (eg. from Excel).

First column should be the old name, second column the new name.

Add

Regex mode off
help
 Clear

#### Apply Show / Hide Samples

Hide matching samples

Show only matching samples

+

Regex mode off
help
 Clear

#### Export Plots

- Images
- Data

px

px

Aspect ratio

PNG
JPEG
SVG

Plot scaling

X

Download the raw data used to create the plots in this report below:

Format:

Tab-separated
Comma-separated
JSON

Note that additional data was saved in `multiqc_data` when this report was generated.

---

##### Choose Plots

 All
 None

---


   Download Plot Images

If you use plots from MultiQC in a publication or presentation, please cite:

> **MultiQC: Summarize analysis results for multiple tools and samples in a single report**  
> *Philip Ewels, Måns Magnusson, Sverker Lundin and Max Käller*  
> Bioinformatics (2016)  
> doi: 10.1093/bioinformatics/btw354  
> PMID: 27312411

#### Save Settings

You can save the toolbox settings for this report to the browser.

 Save


---

#### Load Settings

Choose a saved report profile from the dropdown box below:

[ select ]

Load
 Delete

#### About MultiQC

This report was generated using MultiQC, version 1.0.dev0

You can see a YouTube video describing how to use MultiQC reports here:
https://youtu.be/qPbIlO\_KWN0

For more information about MultiQC, including other videos and
extensive documentation, please visit http://multiqc.info

You can report bugs, suggest improvements and find the source code for MultiQC on GitHub:
https://github.com/ewels/MultiQC

MultiQC is published in Bioinformatics:

> **MultiQC: Summarize analysis results for multiple tools and samples in a single report**  
> *Philip Ewels, Måns Magnusson, Sverker Lundin and Max Käller*  
> Bioinformatics (2016)  
> doi: 10.1093/bioinformatics/btw354  
> PMID: 27312411

# 

A modular tool to aggregate results from bioinformatics analyses across many samples into a single report.

Report generated on 2021-09-01, 04:09 based on data in:
`/mnt/lexnas09/Services/slamdunk/data/300564_Robert_Hunter_SLAM-QS_Liver/slamdunk_out.quantseq_fwd_trim`

---

×
don't show again

**Welcome!** Not sure where to start?  
Watch a tutorial video
  *(6:06)*

## General Statistics

 Copy table

 Sort by highlight
Showing 24/24 rows and 4/4 columns.

| Sample Name | Counted | Retained | Mapped | Sequenced |
| --- | --- | --- | --- | --- |
| 7017\_TCTTAA\_5017\_CAATTC | 10.90 M | 18.79 M | 22.89 M | 24.04 M |
| 7018\_GTCAGG\_5018\_CGGAAT | 9.36 M | 17.87 M | 21.96 M | 22.77 M |
| 7019\_ATACTG\_5019\_GCGCAG | 9.54 M | 16.46 M | 20.28 M | 21.28 M |
| 7020\_TATGTC\_5020\_TTTTCG | 10.54 M | 19.04 M | 23.02 M | 24.04 M |
| 7021\_GAGTCC\_5021\_ACACGA | 10.15 M | 17.31 M | 21.24 M | 22.06 M |
| 7022\_GGAGGT\_5022\_TGTACT | 10.68 M | 18.31 M | 22.81 M | 23.94 M |
| 7023\_CACACT\_5023\_GACGTC | 9.26 M | 16.28 M | 20.06 M | 21.04 M |
| 7024\_CCGCAA\_5024\_ATCGGA | 9.11 M | 17.59 M | 21.88 M | 22.81 M |
| 7025\_TTTATG\_5025\_GGACTC | 9.32 M | 17.66 M | 21.38 M | 22.45 M |
| 7026\_AACGCC\_5026\_TAGTCT | 10.26 M | 18.18 M | 22.16 M | 22.92 M |
| 7027\_CAAGCA\_5027\_ATTAAT | 10.14 M | 18.73 M | 23.03 M | 23.91 M |
| 7028\_GCTCGA\_5028\_TAGGGA | 10.14 M | 18.60 M | 22.71 M | 23.89 M |
| 7029\_GCGAAT\_5029\_ACCGAG | 10.23 M | 15.61 M | 18.46 M | 19.59 M |
| 7030\_TGGATT\_5030\_CCTCTC | 10.19 M | 16.66 M | 21.18 M | 22.17 M |
| 7031\_ACCTAC\_5031\_CTAAGG | 12.85 M | 18.96 M | 22.31 M | 23.21 M |
| 7032\_CGAAGG\_5032\_GGGTCG | 9.95 M | 15.32 M | 18.94 M | 19.91 M |
| 7033\_AGATAG\_5033\_CCACAT | 10.70 M | 15.61 M | 19.59 M | 20.52 M |
| 7034\_TTGGTA\_5034\_GTTGAG | 10.07 M | 15.48 M | 19.40 M | 20.63 M |
| 7035\_GTTACC\_5035\_AGGAGA | 9.49 M | 14.75 M | 18.89 M | 20.02 M |
| 7036\_CGCAAC\_5036\_CAGCCG | 9.09 M | 14.02 M | 17.89 M | 19.01 M |
| 7037\_TGGCGA\_5037\_AAAGGC | 9.58 M | 14.56 M | 18.72 M | 19.73 M |
| 7038\_ACCGTG\_5038\_TGCGTA | 10.66 M | 15.31 M | 19.12 M | 19.95 M |
| 7039\_CAACAG\_5039\_TATATC | 9.54 M | 13.61 M | 17.78 M | 19.14 M |
| 7040\_GATTGT\_5040\_GCTTCT | 10.83 M | 15.59 M | 20.46 M | 21.60 M |

×

#### General Statistics: Columns

Uncheck the tick box to hide columns. Click and drag the handle on the left to change order.

Show All
Show None

| Sort | Visible | Group | Column | Description | ID | Scale |
| --- | --- | --- | --- | --- | --- | --- |
| || |  | Slamdunk | Counted | # reads counted within 3'UTRs | `counted` | read\_count |
| || |  | Slamdunk | Retained | # retained reads after filtering | `retained` | read\_count |
| || |  | Slamdunk | Mapped | # mapped reads | `mapped` | read\_count |
| || |  | Slamdunk | Sequenced | # sequenced reads | `sequenced` | read\_count |

Close

## Slamdunk

Slamdunk is a tool to analyze SLAMSeq data.

### Filter statistics

This table shows the number of reads filtered with each filter criterion during filtering phase of slamdunk.

 Copy table

 Sort by highlight
Showing 24/24 rows and 5/5 columns.

| Sample Name | Mapped | Multimap-Filtered | NM-Filtered | Identity-Filtered | MQ-Filtered |
| --- | --- | --- | --- | --- | --- |
| 7017\_TCTTAA\_5017\_CAATTC | 22.89 M | 3.21 M | 0.00 M | 0.89 M | 0.00 M |
| 7018\_GTCAGG\_5018\_CGGAAT | 21.96 M | 3.51 M | 0.00 M | 0.58 M | 0.00 M |
| 7019\_ATACTG\_5019\_GCGCAG | 20.28 M | 3.01 M | 0.00 M | 0.81 M | 0.00 M |
| 7020\_TATGTC\_5020\_TTTTCG | 23.02 M | 3.16 M | 0.00 M | 0.82 M | 0.00 M |
| 7021\_GAGTCC\_5021\_ACACGA | 21.24 M | 3.26 M | 0.00 M | 0.67 M | 0.00 M |
| 7022\_GGAGGT\_5022\_TGTACT | 22.81 M | 3.68 M | 0.00 M | 0.83 M | 0.00 M |
| 7023\_CACACT\_5023\_GACGTC | 20.06 M | 2.96 M | 0.00 M | 0.82 M | 0.00 M |
| 7024\_CCGCAA\_5024\_ATCGGA | 21.88 M | 3.51 M | 0.00 M | 0.77 M | 0.00 M |
| 7025\_TTTATG\_5025\_GGACTC | 21.38 M | 2.77 M | 0.00 M | 0.95 M | 0.00 M |
| 7026\_AACGCC\_5026\_TAGTCT | 22.16 M | 3.36 M | 0.00 M | 0.63 M | 0.00 M |
| 7027\_CAAGCA\_5027\_ATTAAT | 23.03 M | 3.65 M | 0.00 M | 0.65 M | 0.00 M |
| 7028\_GCTCGA\_5028\_TAGGGA | 22.71 M | 3.08 M | 0.00 M | 1.02 M | 0.00 M |
| 7029\_GCGAAT\_5029\_ACCGAG | 18.46 M | 1.98 M | 0.00 M | 0.87 M | 0.00 M |
| 7030\_TGGATT\_5030\_CCTCTC | 21.18 M | 3.35 M | 0.00 M | 1.17 M | 0.00 M |
| 7031\_ACCTAC\_5031\_CTAAGG | 22.31 M | 2.72 M | 0.00 M | 0.63 M | 0.00 M |
| 7032\_CGAAGG\_5032\_GGGTCG | 18.94 M | 2.73 M | 0.00 M | 0.90 M | 0.00 M |
| 7033\_AGATAG\_5033\_CCACAT | 19.59 M | 3.18 M | 0.00 M | 0.80 M | 0.00 M |
| 7034\_TTGGTA\_5034\_GTTGAG | 19.40 M | 2.82 M | 0.00 M | 1.10 M | 0.00 M |
| 7035\_GTTACC\_5035\_AGGAGA | 18.89 M | 2.92 M | 0.00 M | 1.22 M | 0.00 M |
| 7036\_CGCAAC\_5036\_CAGCCG | 17.89 M | 2.72 M | 0.00 M | 1.15 M | 0.00 M |
| 7037\_TGGCGA\_5037\_AAAGGC | 18.72 M | 3.19 M | 0.00 M | 0.97 M | 0.00 M |
| 7038\_ACCGTG\_5038\_TGCGTA | 19.12 M | 3.19 M | 0.00 M | 0.63 M | 0.00 M |
| 7039\_CAACAG\_5039\_TATATC | 17.78 M | 2.75 M | 0.00 M | 1.42 M | 0.00 M |
| 7040\_GATTGT\_5040\_GCTTCT | 20.46 M | 3.73 M | 0.00 M | 1.13 M | 0.00 M |

×

#### Slamdunk Filtering Table: Columns

Uncheck the tick box to hide columns. Click and drag the handle on the left to change order.

Show All
Show None

| Sort | Visible | Group | Column | Description | ID | Scale |
| --- | --- | --- | --- | --- | --- | --- |
| || |  | Slamdunk | Mapped | # mapped reads | `mapped` | read\_count |
| || |  | Slamdunk | Multimap-Filtered | # multimap-filtered reads | `multimapper` | read\_count |
| || |  | Slamdunk | NM-Filtered | # NM-filtered reads | `nmfiltered` | read\_count |
| || |  | Slamdunk | Identity-Filtered | # identity-filtered reads | `idfiltered` | read\_count |
| || |  | Slamdunk | MQ-Filtered | # MQ-filtered reads | `mqfiltered` | read\_count |

Close

---

### PCA (T>C based)

This plot shows the principal components of samples based
on the distribution of reads with T>C conversions within UTRs
(see the slamdunk docs).

loading..

---

### Conversion rates per UTR

This plot shows the individual conversion rates for all UTRs
(see the slamdunk docs).

loading..

---

### Conversion rates per read

This plot shows the individual conversion rates over all reads.
It shows these conversion rates strand-specific: This means for a properly labelled
sample you would see a T>C excess on the plus-strand and an A>G excess on the minus strand
(see the slamdunk docs).

Plus Strand +
Minus Strand -

loading..

---

### Non T>C mutations over read positions

This plot shows the distribution of non T>C mutations across read positions
(see the slamdunk docs).

Forward reads +
Reverse reads -

loading..

---

### T>C conversions over read positions

This plot shows the distribution of T>C conversions across read positions
(see the slamdunk docs).

Forward reads +
Reverse reads -

loading..

---

### Non T>C mutations over UTR positions

This plot shows the distribution of non T>C mutations across UTR positions for the last 200 bp from the 3' UTR end
(see the slamdunk docs).

UTRs on plus strand
UTRs on minus strand

loading..

---

### T>C conversions over UTR positions

This plot shows the distribution of T>C conversions across UTR positions for the last 200 bp from the 3' UTR end
(see the slamdunk docs).

UTRs on plus strand
UTRs on minus strand

loading..

**MultiQC v1.0.dev0**
- Written by Phil Ewels,
available on GitHub.

This report uses HighCharts,
jQuery,
jQuery UI,
Bootstrap,
chroma.js,
FileSaver.js and
clipboard.js.

×

### Plot Table Data

Select Column

Select Column

Please select two table columns.

Close

×

### Regex Help

Toolbox search strings can behave as regular expressions (regexes). Click a button below to see an example of it in action. Try modifying them yourself in the text box.

`^` (start of string)
`$` (end of string)
`[]` (character choice)
`\d` (shorthand for `[0-9]`)
`\w` (shorthand for `[0-9a-zA-Z_]`)
`.` (any character)
`\.` (literal full stop)
`()` `|` (group / separator)
`*` (prev char 0 or more)
`+` (prev char 1 or more)
`?` (prev char 0 or 1)
`{}` (char num times)
`{,}` (count range)

```
samp_1
samp_1_edited
samp_2
samp_2_edited
samp_3
samp_3_edited
prepended_samp_1
tmp_samp_1_edited
tmpp_samp_1_edited
tmppp_samp_1_edited
#samp_1_edited.tmp
samp_11
samp_11111
```

See regex101.com for a more heavy duty testing suite.

Close
