# Supplementary material for: SLAMseq reveals potential transfer of RNA from liver to kidney in the mouse
Source: Nat Commun. 2025 Aug 11;16:7413. doi: 10.1038/s41467-025-62688-9 (PMC12339719; doi:10.1038/s41467-025-62688-9)
Supplement: Supplementary file 6 — Source Data [file 41467_2025_62688_MOESM6_ESM.zip › Source data/FigS3a_uncropped.pdf]

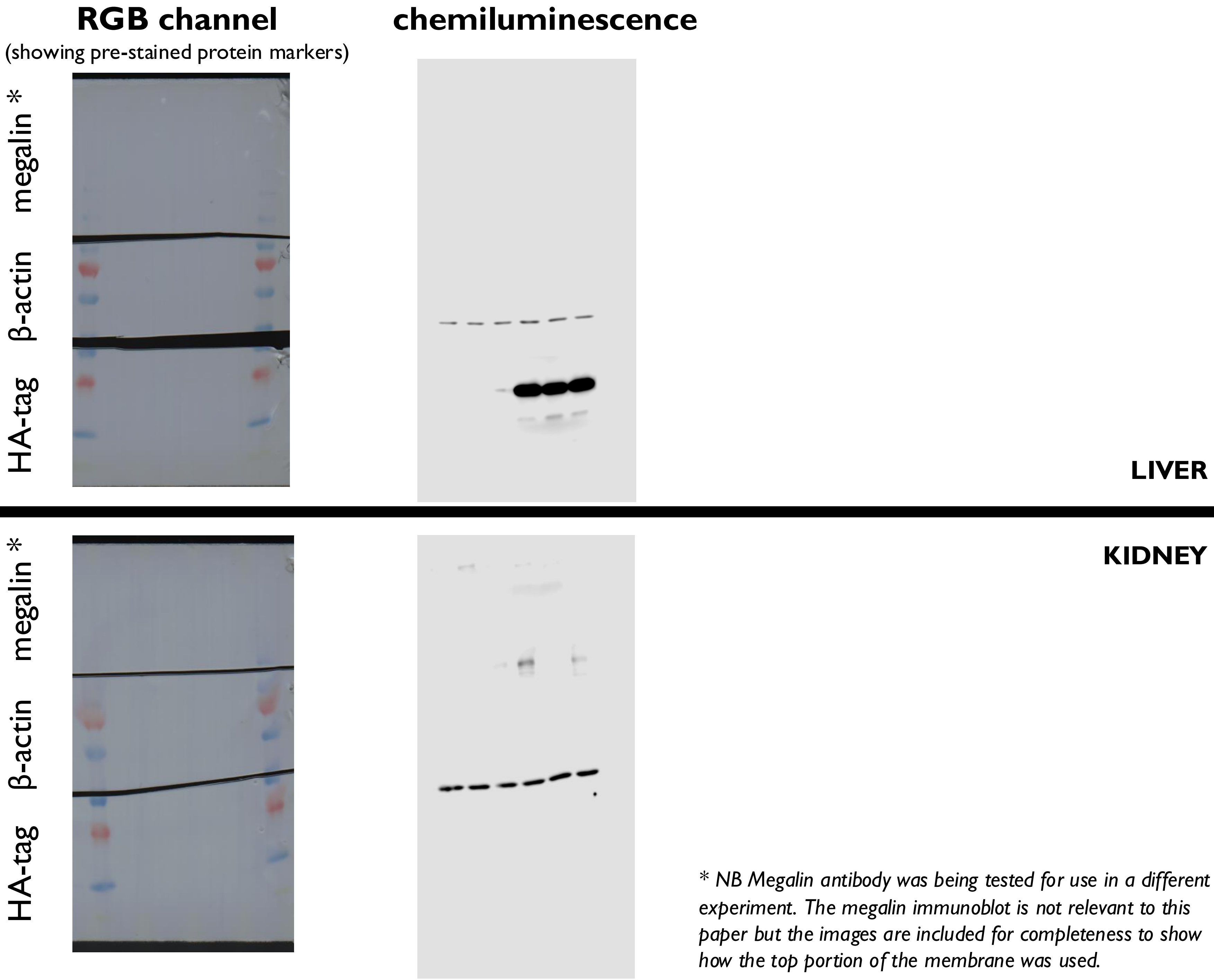

** NB Megalin antibody was being tested for use in a different experiment. The megalin immunoblot is not relevant to this paper but the images are included for completeness to show how the top portion of the membrane was used.*
