# Supplementary figures and images for: SLAMseq reveals potential transfer of RNA from liver to kidney in the mouse
Source: Nat Commun. 2025 Aug 11;16:7413. doi: 10.1038/s41467-025-62688-9 (PMC12339719; doi:10.1038/s41467-025-62688-9)

DOTBLT 04/04/23

ROB HUNTER

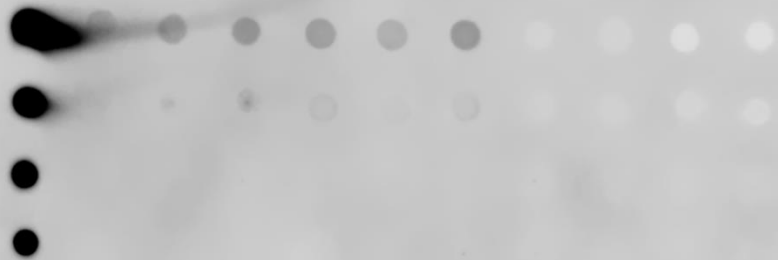

Supplement: Supplementary file 6 — Source Data [file 41467_2025_62688_MOESM6_ESM.zip › Source data/Fig2a_uncropped.pdf]

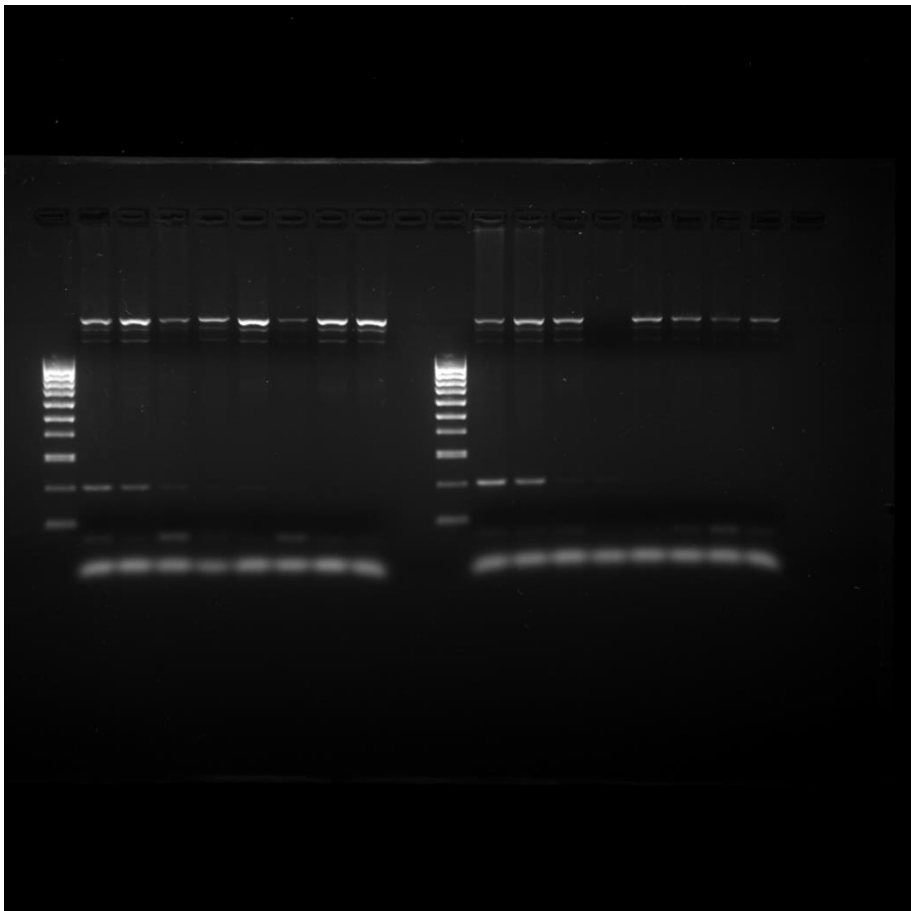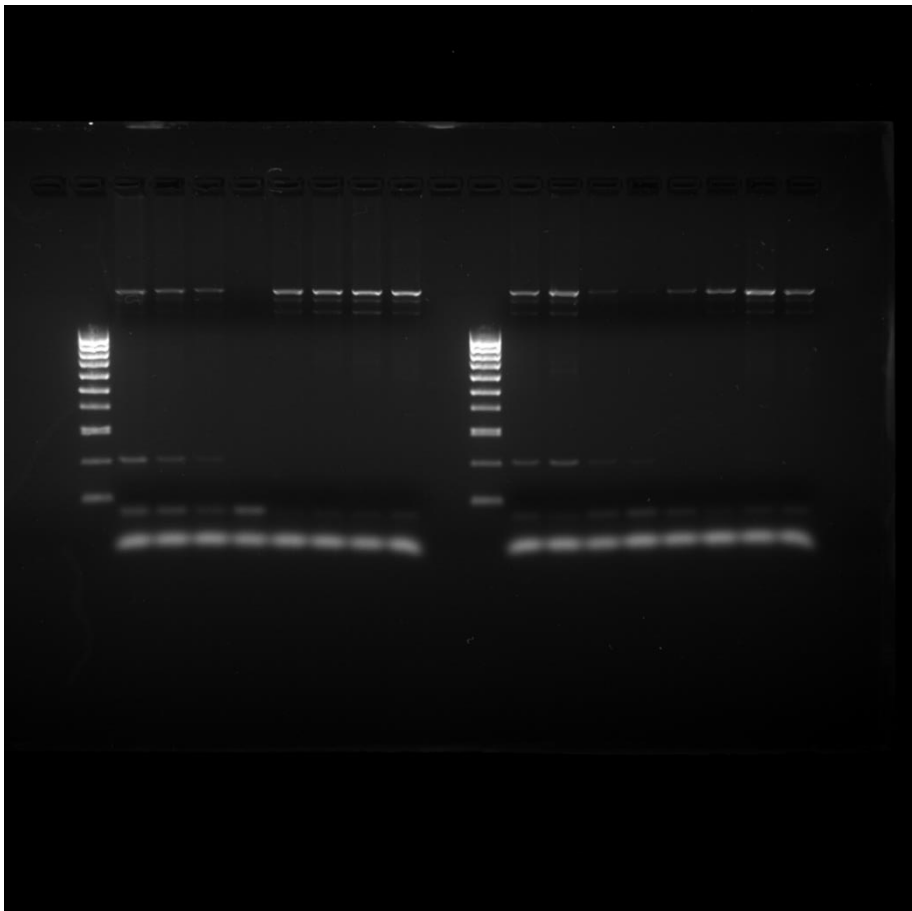

Supplement: Supplementary file 6 — Source Data [file 41467_2025_62688_MOESM6_ESM.zip › Source data/FigS2b_uncropped.pdf]

# LIVER

# KIDNEY

Cre +ve

-ve

Cre +ve

-ve

N

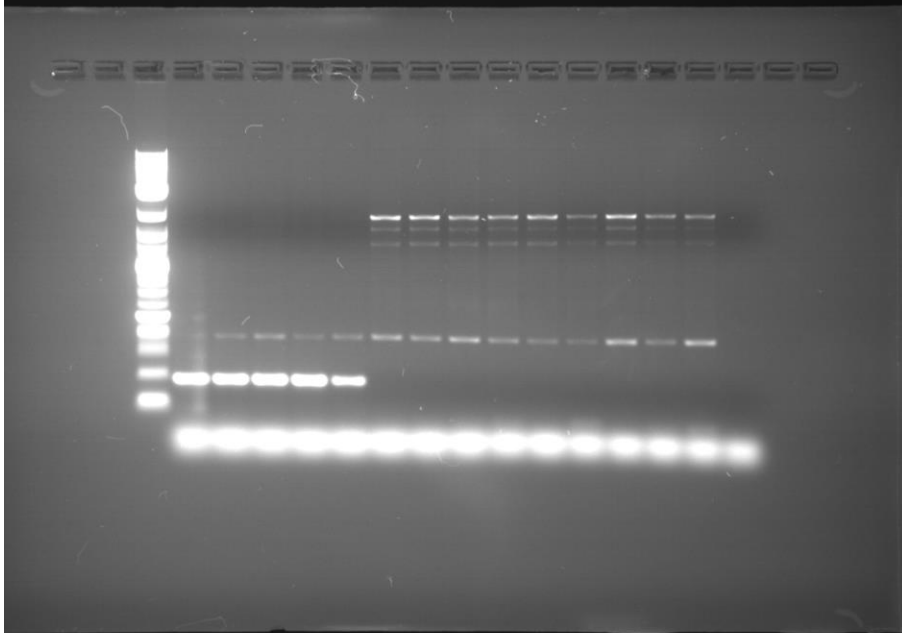

N = no template control

# HEART

# SPLEEN

Cre +ve

-ve

Cre +ve

-ve

N

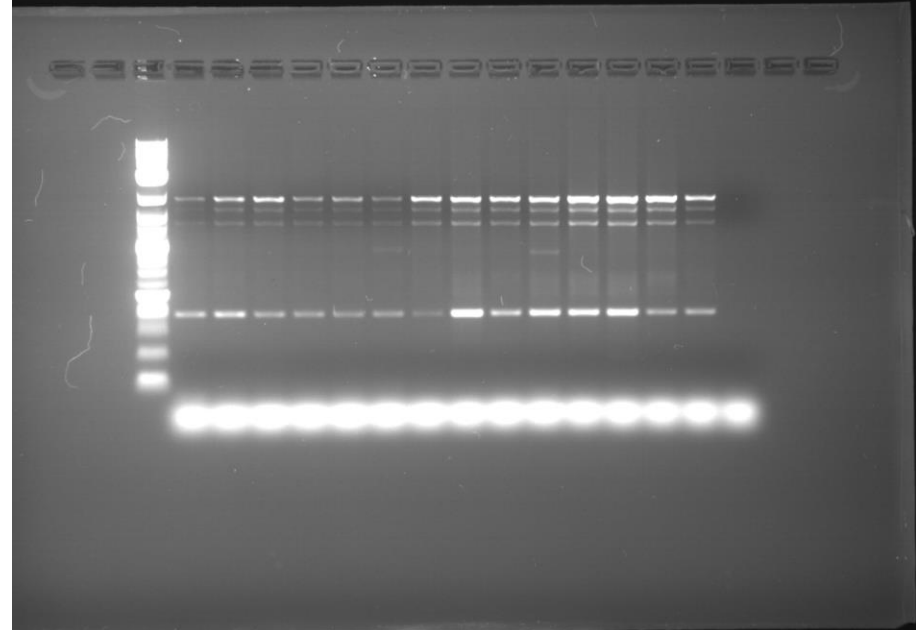

Supplement: Supplementary file 6 — Source Data [file 41467_2025_62688_MOESM6_ESM.zip › Source data/Fig1a_uncropped.pdf]
