# Supplementary material for: SLAMseq reveals potential transfer of RNA from liver to kidney in the mouse
Source: Nat Commun. 2025 Aug 11;16:7413. doi: 10.1038/s41467-025-62688-9 (PMC12339719; doi:10.1038/s41467-025-62688-9)
Supplement: Supplementary file 3 — Supplementary Data 1-13 [file 41467_2025_62688_MOESM3_ESM.zip › Supplementary file 3.zip!/injury_expt_kidney_mRNA_multiqc_report.html]

MultiQC Report


# Toggle navigation v1.5 (fb8cb83)

Loading report..

- General Stats
- Slamdunk
  - Filter statistics
  - PCA (T>C based)
  - Conversion rates per UTR
  - Conversion rates per read
  - Non T>C mismatches over read positions
  - T>C conversions over read positions
  - Non T>C mismatches over UTR positions
  - T>C conversions over UTR positions

Toolbox

### MultiQC Toolbox

#### Apply Highlight Samples

This report has flat image plots that won't be highlighted.

+

Regex mode off
help
 Clear

#### Apply Rename Samples

This report has flat image plots that won't be renamed.  
See the documentation
for help.

+

Click here for bulk input.

Paste two columns of a tab-delimited table here (eg. from Excel).

First column should be the old name, second column the new name.

Add

Regex mode off
help
 Clear

#### Apply Show / Hide Samples

This report has flat image plots that won't be hidden.  
See the documentation
for help.

Hide matching samples

Show only matching samples

+

Regex mode off
help
 Clear

#### Export Plots

- Images
- Data

px

px

Aspect ratio

PNG
JPEG
SVG

Plot scaling

X

Download the raw data used to create the plots in this report below:

Format:

Tab-separated
Comma-separated
JSON

Note that additional data was saved in `multiqc_data` when this report was generated.

---

##### Choose Plots

 All
 None


---


   Download Plot Images

If you use plots from MultiQC in a publication or presentation, please cite:

> **MultiQC: Summarize analysis results for multiple tools and samples in a single report**  
> *Philip Ewels, Måns Magnusson, Sverker Lundin and Max Käller*  
> Bioinformatics (2016)  
> doi: 10.1093/bioinformatics/btw354  
> PMID: 27312411

#### Save Settings

You can save the toolbox settings for this report to the browser.

 Save


---

#### Load Settings

Choose a saved report profile from the dropdown box below:

[ select ]

Load
 Delete
 Set default
 Clear default

#### About MultiQC

This report was generated using MultiQC, version 1.5 (fb8cb83)

You can see a YouTube video describing how to use MultiQC reports here:
https://youtu.be/qPbIlO\_KWN0

For more information about MultiQC, including other videos and
extensive documentation, please visit http://multiqc.info

You can report bugs, suggest improvements and find the source code for MultiQC on GitHub:
https://github.com/ewels/MultiQC

MultiQC is published in Bioinformatics:

> **MultiQC: Summarize analysis results for multiple tools and samples in a single report**  
> *Philip Ewels, Måns Magnusson, Sverker Lundin and Max Käller*  
> Bioinformatics (2016)  
> doi: 10.1093/bioinformatics/btw354  
> PMID: 27312411

A modular tool to aggregate results from bioinformatics analyses across many samples into a single report.

#### JavaScript Disabled

MultiQC reports use JavaScript for plots and toolbox functions. It looks like
you have JavaScript disabled in your web browser. Please note that many of the report
functions will not work as intended.

Loading report..

Report generated on 2023-08-15, 01:25 based on data in:
`/home/data/DAP/DEV/2935541/tmp`

---

×
don't show again

**Welcome!** Not sure where to start?  
Watch a tutorial video
  *(6:06)*

## General Statistics

 Copy table

 Configure Columns

 Sort by highlight

 Plot
Showing ^24^/_24_ rows and ^4^/_4_ columns.

| Sample Name | M Counted | M Retained | M Mapped | M Sequenced |
| --- | --- | --- | --- | --- |
| sample\_25 | 13.24 | 21.21 | 25.81 | 26.18 |
| sample\_26 | 11.81 | 19.92 | 24.28 | 24.66 |
| sample\_27 | 14.63 | 23.94 | 28.65 | 29.14 |
| sample\_28 | 14.17 | 21.87 | 26.22 | 26.51 |
| sample\_29 | 16.21 | 25.27 | 30.10 | 30.43 |
| sample\_30 | 15.63 | 24.65 | 29.57 | 29.93 |
| sample\_31 | 14.84 | 23.06 | 27.71 | 28.11 |
| sample\_32 | 13.32 | 21.26 | 25.64 | 26.27 |
| sample\_33 | 13.71 | 21.83 | 26.04 | 26.69 |
| sample\_34 | 19.21 | 30.40 | 36.08 | 36.54 |
| sample\_35 | 13.73 | 22.78 | 27.34 | 27.70 |
| sample\_36 | 16.18 | 27.33 | 33.10 | 33.55 |
| sample\_37 | 14.35 | 24.37 | 30.00 | 30.47 |
| sample\_38 | 11.89 | 19.35 | 23.66 | 24.00 |
| sample\_39 | 14.25 | 22.48 | 27.08 | 27.44 |
| sample\_40 | 14.44 | 22.91 | 27.54 | 28.03 |
| sample\_41 | 11.76 | 19.61 | 23.40 | 23.65 |
| sample\_42 | 13.12 | 20.92 | 25.41 | 25.80 |
| sample\_43 | 12.22 | 20.11 | 24.49 | 24.79 |
| sample\_44 | 10.72 | 17.77 | 21.09 | 21.60 |
| sample\_45 | 11.93 | 19.83 | 24.33 | 24.63 |
| sample\_46 | 13.42 | 22.11 | 26.98 | 27.34 |
| sample\_47 | 13.44 | 21.94 | 26.15 | 26.46 |
| sample\_48 | 12.23 | 19.10 | 22.62 | 22.97 |

×

#### General Statistics: Columns

Uncheck the tick box to hide columns. Click and drag the handle on the left to change order.

Show All
Show None

| Sort | Visible | Group | Column | Description | ID | Scale |
| --- | --- | --- | --- | --- | --- | --- |
| \|\| |  | Slamdunk | M Counted | # reads counted within 3'UTRs (millions) | `counted` | read\_count |
| \|\| |  | Slamdunk | M Retained | # retained reads after filtering (millions) | `retained` | read\_count |
| \|\| |  | Slamdunk | M Mapped | # mapped reads (millions) | `mapped` | read\_count |
| \|\| |  | Slamdunk | M Sequenced | # sequenced reads (millions) | `sequenced` | read\_count |

Close

## Slamdunk

Slamdunk is a tool to analyze SLAMSeq data.

### Filter statistics

This table shows the number of reads filtered with each filter criterion during filtering phase of slamdunk.

Copy table

 Configure Columns

 Sort by highlight

 Plot
Showing ^24^/_24_ rows and ^5^/_5_ columns.

| Sample Name | M Mapped | M Multimap-Filtered | M NM-Filtered | M Identity-Filtered | M MQ-Filtered |
| --- | --- | --- | --- | --- | --- |
| sample\_25 | 25.81M | 4.46M | 0.00M | 0.14M | 0.00M |
| sample\_26 | 24.28M | 4.22M | 0.00M | 0.14M | 0.00M |
| sample\_27 | 28.65M | 4.53M | 0.00M | 0.18M | 0.00M |
| sample\_28 | 26.22M | 4.19M | 0.00M | 0.16M | 0.00M |
| sample\_29 | 30.10M | 4.63M | 0.00M | 0.19M | 0.00M |
| sample\_30 | 29.57M | 4.74M | 0.00M | 0.18M | 0.00M |
| sample\_31 | 27.71M | 4.48M | 0.00M | 0.16M | 0.00M |
| sample\_32 | 25.64M | 4.22M | 0.00M | 0.16M | 0.00M |
| sample\_33 | 26.04M | 4.05M | 0.00M | 0.17M | 0.00M |
| sample\_34 | 36.08M | 5.44M | 0.00M | 0.23M | 0.00M |
| sample\_35 | 27.34M | 4.39M | 0.00M | 0.16M | 0.00M |
| sample\_36 | 33.10M | 5.57M | 0.00M | 0.20M | 0.00M |
| sample\_37 | 30.00M | 5.45M | 0.00M | 0.18M | 0.00M |
| sample\_38 | 23.66M | 4.16M | 0.00M | 0.15M | 0.00M |
| sample\_39 | 27.08M | 4.44M | 0.00M | 0.16M | 0.00M |
| sample\_40 | 27.54M | 4.46M | 0.00M | 0.16M | 0.00M |
| sample\_41 | 23.40M | 3.65M | 0.00M | 0.13M | 0.00M |
| sample\_42 | 25.41M | 4.33M | 0.00M | 0.16M | 0.00M |
| sample\_43 | 24.49M | 4.22M | 0.00M | 0.16M | 0.00M |
| sample\_44 | 21.09M | 3.16M | 0.00M | 0.16M | 0.00M |
| sample\_45 | 24.33M | 4.38M | 0.00M | 0.13M | 0.00M |
| sample\_46 | 26.98M | 4.71M | 0.00M | 0.16M | 0.00M |
| sample\_47 | 26.15M | 4.04M | 0.00M | 0.17M | 0.00M |
| sample\_48 | 22.62M | 3.38M | 0.00M | 0.14M | 0.00M |

×

#### Slamdunk Filtering Table: Columns

Uncheck the tick box to hide columns. Click and drag the handle on the left to change order.

Show All
Show None

| Sort | Visible | Group | Column | Description | ID | Scale |
| --- | --- | --- | --- | --- | --- | --- |
| \|\| |  | Slamdunk | M Mapped | # mapped reads (millions) | `mapped` | read\_count |
| \|\| |  | Slamdunk | M Multimap-Filtered | # multimap-filtered reads (millions) | `multimapper` | read\_count |
| \|\| |  | Slamdunk | M NM-Filtered | # NM-filtered reads (millions) | `nmfiltered` | read\_count |
| \|\| |  | Slamdunk | M Identity-Filtered | # identity-filtered reads (millions) | `idfiltered` | read\_count |
| \|\| |  | Slamdunk | M MQ-Filtered | # MQ-filtered reads (millions) | `mqfiltered` | read\_count |

Close

---

### PCA (T>C based)

This plot shows the principal components of samples based
on the distribution of reads with T>C conversions within UTRs
(see the slamdunk docs).

loading..

---

### Conversion rates per UTR

This plot shows the individual conversion rates for all UTRs
(see the slamdunk docs).

loading..

---

### Conversion rates per read

This plot shows the individual conversion rates over all reads.
It shows these conversion rates strand-specific: This means for a properly labeled
sample you would see a T>C excess on the plus-strand and an A>G excess on the minus strand
(see the slamdunk docs).

Plus Strand +
Minus Strand -

loading..

---

### Non T>C mismatches over read positions

This plot shows the distribution of non T>C mismatches across read positions
(see the slamdunk docs).

Forward reads +
Reverse reads -

loading..

---

### T>C conversions over read positions

This plot shows the distribution of T>C conversions across read positions
(see the slamdunk docs).

Forward reads +
Reverse reads -

loading..

---

### Non T>C mismatches over UTR positions

This plot shows the distribution of non T>C mismatches across UTR positions for the last 250 bp from the 3' UTR end
(see the slamdunk docs).

UTRs on plus strand
UTRs on minus strand

loading..

---

### T>C conversions over UTR positions

This plot shows the distribution of T>C conversions across UTR positions for the last 250 bp from the 3' UTR end
(see the slamdunk docs).

UTRs on plus strand
UTRs on minus strand

loading..

**MultiQC v1.5 (fb8cb83)** 
- Written by Phil Ewels,
available on GitHub.

This report uses HighCharts,
jQuery,
jQuery UI,
Bootstrap,
FileSaver.js and
clipboard.js.

×

### Plot Table Data

Select Column

Select Column

Please select two table columns.

Close

×

### Regex Help

Toolbox search strings can behave as regular expressions (regexes). Click a button below to see an example of it in action. Try modifying them yourself in the text box.

`^` (start of string)
`$` (end of string)
`[]` (character choice)
`\d` (shorthand for `[0-9]`)
`\w` (shorthand for `[0-9a-zA-Z_]`)
`.` (any character)
`\.` (literal full stop)
`()` `|` (group / separator)
`*` (prev char 0 or more)
`+` (prev char 1 or more)
`?` (prev char 0 or 1)
`{}` (char num times)
`{,}` (count range)

```
samp_1
samp_1_edited
samp_2
samp_2_edited
samp_3
samp_3_edited
prepended_samp_1
tmp_samp_1_edited
tmpp_samp_1_edited
tmppp_samp_1_edited
#samp_1_edited.tmp
samp_11
samp_11111
```

See regex101.com for a more heavy duty testing suite.

Close
